# Associations between adherence to public health measures and changes in alcohol consumption among middle-aged and older adults during the COVID-19 pandemic: the Canadian Longitudinal Study on Aging (CLSA)

**DOI:** 10.24095/hpcdp.46.1.03

**Published:** 2026-01

**Authors:** Kiara Pannozzo, Lauren E. Griffith, Aaron Jones, Vanessa De Rubeis, Jayati Khattar, Margaret de Groh, Ying Jiang, Jacqueline McMillan, Laura N. Anderson

**Affiliations:** 1 Department of Health Research Methods, Evidence, and Impact, McMaster University, Hamilton, Ontario, Canada; 2 Centre for Surveillance and Applied Research, Public Health Agency of Canada, Ottawa, Ontario, Canada; 3 Division of Geriatric Medicine, Cumming School of Medicine, University of Calgary, Calgary, Alberta, Canada

**Keywords:** CLSA, COVID-19, alcohol use change, public health measure, adherence, alcohol consumption

## Abstract

**Introduction:**

The COVID-19 pandemic and associated public health measures (PHMs) potentially affected alcohol consumption. Our objectives were to evaluate if adherence to PHMs was associated with changes in alcohol consumption and binge drinking during the COVID-19 pandemic.

**Methods:**

A prospective cohort study was conducted with participants (50–96 years) in the Canadian Longitudinal Study on Aging (N=23615). Adjusted odds ratios (aORs) were estimated from multinomial logistic regression models for associations between PHM adherence (self-quarantine, attending public gatherings, leaving home, mask wearing and handwashing) and self-reported changes in alcohol consumption during the first year of the pandemic and prospectively measured changes in alcohol consumption frequency and frequency of binge-drinking events from 2015–2018 to 2020.

**Results:**

During the first year of the pandemic, 13% (n=2733) of participants self-reported increased alcohol consumption, while 13% (n = 2921) self-reported decreased consumption. Prospective measures suggested 19.1% (n = 4421) increased and 34.5% (n = 7971) decreased consumption frequency, while 12.9% (n = 1427) increased and 17.6% (n = 1953) decreased frequency of binge-drinking events. High PHM adherence, compared to low, was associated with higher odds of decreased alcohol consumption frequency (aOR = 1.17; 95% confidence interval [CI]: 1.06–1.30). No associations were observed between PHM adherence and self-reported change in alcohol consumption or frequency of binge-drinking events. Associations were consistent across socioeconomic groups.

**Conclusion:**

PHM adherence was associated with decreased, and not increased, frequency of alcohol consumption by adults aged 50–96 years in the first year of the COVID-19 pandemic.

**Highlights**

- We examined the association between adherence to the public health measures initiated to limit COVID- 19 spread and self-reported and prospective changes in frequency of alcohol consumption and binge drinking by adults aged 50 to 96 years.
- Up to 20% reported increasing their alcohol consumption and frequency of binge drinking or of alcohol consumption.
- Up to 35% reported decreasing frequency of their alcohol consumption.
- Greater adherence to public health measures appeared to be associated with higher odds of decreasing frequency of alcohol consumption.
- Adherence to the public health measures did not result in increased alcohol consumption by middleaged and older adults.

## Introduction

At the onset of the COVID-19 pandemic, border closures, school closures, business operation restrictions, quarantine and stay-at-home orders, and other public health measures (PHMs) were implemented to limit non-essential social interactions and minimize COVID-19 transmission, deaths and strain on health care systems.^1^-^3^ While PHMs were essential for slowing down SARS-CoV-2 transmission, the restrictions resulted in job losses and reduced incomes, introduced uncertainty and increased stress levels.^1^,^2^

Despite limited social activities, alcohol consumption and sales increased in Canada after the onset of the pandemic.^4^,^5^ Given that research has identified links between alcohol use and coping strategies,^6^ an increase in consumption may have been a reflection of increased stress.^4^,^7^ Research investigating the effects of disasters^8^ and quarantine^9^ have found strong associations between psychological stress and increased substance use. Job and income loss can also increase stress levels, which can, in turn, lead to increased alcohol consumption and related health concerns.^10^ The pandemic may have also disproportionately impacted Canadians aged 65 years and older as they experienced most of the excess deaths and may have been at greater risk for social isolation.^11^

Changes in alcohol consumption during the pandemic were most frequently observed among male participants.^7^ Higher rates of increased alcohol consumption were also observed among individuals in higher-income groups, those who were divorced, separated or widowed, those who were unhoused and those aged 60 years and older.^12^,^13^ One report found that 13% of the older adults sampled increased their alcohol consumption during the pandemic, which is a concern due to their heightened sensitivity to alcohol and alcohol-related effects.^14^,^15^

Given the effects of the pandemic on older Canadians^11^ and the adverse health outcomes associated with excess alcohol consumption,^16^ examining alcohol intake in this population is crucial to understanding the impact of the pandemic and pandemic responses, including adherence to PHMs, on changes in alcohol intake. 

Despite the disproportionate effect of the pandemic on equity-deserving groups in Canada, research examining health equity factors on pandemic-related outcomes such as alcohol consumption has shown inconsistent results.^1^

Most of the Canadian studies that reported increased alcohol consumption during the pandemic were cross-sectional and based on self-reported recall of changes in consumption.^4^,^7^,^17^ While some studies evaluated determinants of longitudinal changes in self-reported alcohol consumption among middle-aged and older adults during the pandemic,^15^ none have assessed associations between PHM adherence and changes in alcohol consumption. Further, as individual responses to pandemic-related stress may influence PHM adherence, adherence may affect alcohol consumption.^18^ Because implemented PHMs varied across the provinces, there may also be differences in alcohol consumption changes across Canada.^19^

The objectives of our study were to evaluate the association between longitudinal measures of PHM adherence and self-reported change in alcohol consumption and longitudinal changes (pre-pandemic to early pandemic) in alcohol consumption among adults aged 50 to 96 years, while identifying sociodemographic modifiers of these associations. We hypothesize that increased PHM adherence during the COVID-19 pandemic was associated with increased alcohol consumption as a way to manage the stress associated with increased social isolation and loneliness. 

## Methods

***Study design and setting***

We conducted a longitudinal cohort study with middle-aged and older adults (aged 50–96 years) residing in the 10 Canadian provinces. We report our results in keeping with the Strengthening the Reporting of Observational Studies in Epidemiology (STROBE) guidelines.^20^

***Data source***

We used data collected via the Canadian Longitudinal Study on Aging (CLSA) in this study. The CLSA enrolled participants (45–85 years at the time of recruitment) from the Canadian provinces.^21^ The CLSA includes a Tracking Cohort and a Comprehensive Cohort. Tracking Cohort participants are selected randomly from all 10 provinces and are interviewed via the Internet or over the telephone.^21^,^22^ Comprehensive Cohort participants are selected randomly from within a 20 to 50 km radius of one of 11 data collection sites in seven provinces and are interviewed in person.^21^ Similar information is collected from both cohorts, but Comprehensive Cohort participants undergo a thorough physical assessment.^21^ All eligible participants are cognitively able to independently complete questionnaires in English or French.^21^

At the time of baseline data collections, people living in the territories, on First Nations reserves or in other First Nations settlements, and in institutions as well as full-time members of the Canadian Armed Forces were excluded, in alignment with the Canadian Community Health Survey’s inclusion criteria. 21,23

**Figure 1:**
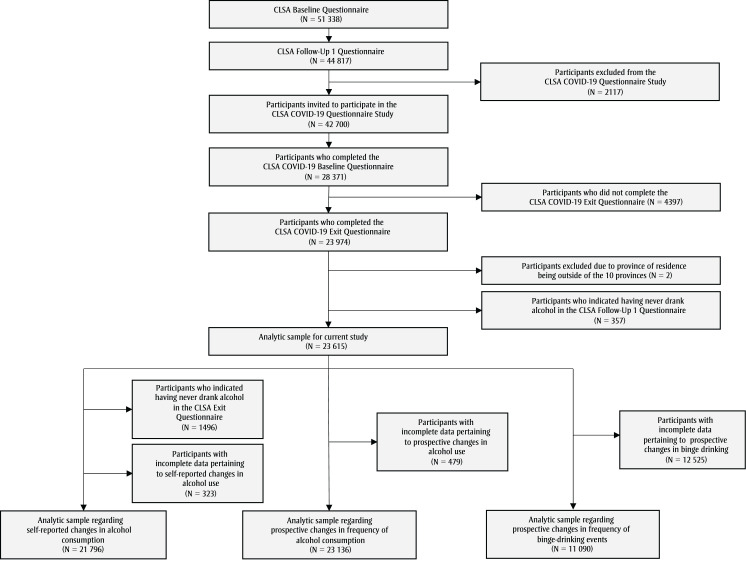
Numbers of participants completing the CLSA Baseline Questionnaire (2011–2015), the Follow-Up 1 Questionnaire (2015–2018) and the COVID-19 Questionnaire Study (2020), Canada

**Abbreviation: **CLSA, Canadian Longitudinal Study on Aging.

Data are collected every 3 years and participants are followed for 20 years (until 2033) or until death or loss to follow-up.^21^ Baseline data were collected between 2011 and 2015, and Follow-Up 1 (FUP1) was conducted between 2015 and 2018.^21^ Of the 51338 participants at baseline, 44817 completed the FUP1 Questionnaire (Figure 1).^21^

The COVID-19 Questionnaire Study assessing the impact of the pandemic was initiated in April 2020.^22^ The study included five questionnaires: the COVID-19 Baseline Questionnaire (conducted between 15 April 2020 and 30 May 2020, and completed by 28559 participants^24^); three questionnaires conducted monthly in July, August and September 2020; and the COVID-19 Exit Questionnaire (conducted between 29 September and 29 December 2020 and completed by 23974 participants).^25^

To ensure accuracy of the analyses on prospective changes in alcohol consumption frequency, self-reported changes in alcohol consumption and prospective changes in frequency of binge-drinking events from FUP1 to completion of the COVID-19 Exit Questionnaire, 357 individuals who indicated that they had never drank alcohol (assessed at FUP1) were excluded from the study as were two respondents who did not reside in the provinces (Figure 1). The final analytic sample included 23615 participants aged 50 to 96 years at the time of taking the CLSA COVID-19 Baseline Questionnaire.

***Ethics approval***

Ethics approval for this study was received from the Hamilton Integrated Research Ethics Board (HiREB #14090).

***Outcome: Measuring alcohol consumption***

We analyzed three outcome measures among respondents who reported ever drinking at FUP1. The first outcome, self-reported changes in alcohol consumption, was measured using the COVID-19 Exit Questionnaire (September to December 2020). Participants who responded “no” to the prompt “Have you ever drank alcohol?” (n = 1496) were excluded from this analysis because they were not asked if their alcohol consumption changed (Figure 1). All participants who responded “yes” were asked, “Since March 1st, 2020, has your alcohol consumption increased, decreased or stayed the same?”

The second outcome, prospective change in the frequency of alcohol consumption, was measured via responses to questions on self-reported alcohol consumption asked pre-pandemic, using the FUP1 Questionnaire (2015–2018), and during the pandemic, using the COVID-19 Exit Questionnaire (September to December 2020). The FUP1 Questionnaire asked about alcohol consumption in the past 12 months, while the COVID-19 Exit Questionnaire asked about alcohol consumption since 1March 2020. The response options were as follows: “never,” “about once a month,” “2 to 3 times a month,” “once a week,” “2 to 3 times a week,” “4 to 5 times a week” and “almost every day.” COVID-19 Exit Questionnaire respondents who indicated that they never drank alcohol were categorized as having no alcohol consumption since 1 March 2020. Based on changes in responses from the FUP1 to the COVID-19 Exit Questionnaires, participants were classified as having increased, decreased or not changed their alcohol consumption. 

The third outcome, prospective change in frequency of binge-drinking events, was measured via responses to questions on self-reported number of binge-drinking events (four or more drinks at the same sitting or occasion for females and five or more drinks at the same sitting or occasion for males) pre-pandemic using the FUP1 Questionnaire and during the pandemic using the COVID-19 Exit Questionnaire. 

The questions about alcohol consumption were adapted from the Ontario Health Study.^26^ Agreement between self-reported changes in alcohol consumption and prospective changes in frequency of alcohol consumption was quantified using the kappa statistic.

***Exposure: Measurement of PHM adherence score***

Data on PHM adherence were collected via the COVID-19 Baseline Questionnaire (April–May 2020) and the three monthly questionnaires. The COVID-19 Baseline Questionnaire and each monthly questionnaire asked participants whether, in the past month, they had been under self-quarantine, attended a large public gathering or left their home for essential reasons (e.g. going to work, buying food, going to a pharmacy or hospital, taking care of dependents) or non-essential reasons (e.g. because they were tired of being inside); on average how many times a day in the past month they had washed their hands; and how often in the past month they had worn a mask when leaving the home (see Supplementary Table 1).^27^

For each PHM, a score between 0 (low adherence) and 1 (high adherence) was assigned. The average individual PHM adherence score at each time point (i.e. at the time of the COVID-19 Baseline Questionnaire and each subsequent monthly questionnaires) for each participant was calculated. An overall PHM adherence score was then calculated for each participant by averaging the scores from each time point. Each PHM was weighted equally across all time points. The overall PHM adherence score was then categorized as low (first quartile of the averaged scores), medium (second and third quartile of the averaged scores) and high (fourth quartile of the averaged scores). This was our primary exposure for regression models. Quartiles were based on the complete COVID-19 baseline sample and applied to the current sample, which excludes nondrinkers and those without COVID-19 Exit Questionnaire data.

***Measurement of sociodemographic characteristics***

Information on participants’ sex, immigrant status, educational attainment and racial background were obtained via the CLSA Baseline Questionnaire; marital status and total household income were obtained via the CLSA FUP1 Questionnaire; and age, region of residence, anxiety symptoms and depression symptoms were obtained via the COVID-19 Baseline Questionnaire. Participants with a score of 10 or more on the 10-item Center for Epidemiologic Studies Depression Scale or the Generalized Anxiety Disorder 7-item screening tool were considered to have symptoms of depression or anxiety, respectively.^28^,^29^

Models were adjusted for the following potential confounders: sex, age at baseline, racial background, marital status, immigrant status, educational attainment, region of residence and total household income. These confounders were selected a priori, as they have been previously associated with the outcome and exposure but not the causal pathway (Supplementary Figure 1).^1^,^12^,^30^-^38^ In addition, sex, age at baseline, marital status, immigrant status and racial background were assessed as equity stratifiers of associations.

***Statistical analysis***

Analysis was completed using statistical package SAS version 9.4 (SAS Institute Inc., Cary, NC, US). For our first objective, we evaluated the association between PHM adherence score and self-reported change in alcohol consumption, prospective changes in frequency of alcohol consumption and prospective changes in frequency of binge-drinking events. We report the percent change in each category (increased, decreased, no change) for each operationalization of the outcome. We used multinomial logistic regression to estimate associations, addressing potential underestimation of standard errors by applying nonparametric bootstrapping with replacements (n = 1000). Unadjusted and adjusted odds ratios (ORs) with associated 95% confidence intervals (CIs) are reported. Variance inflation factors for adjusted models were estimated using a linear regression model to assess multicollinearity. All these factors were less than 5, suggesting multicollinearity was not severe.^39^

For our second objective, we evaluated statistical interactions between overall PHM adherence score and sex, age, marital status, immigrant status and racial background. Stratified results were presented for characteristics with statistically significant interaction terms based on bootstrapped likelihood ratio test *p*values (*p*<0.05) as well as by age and sex. Sampling weights were not available for the COVID-19 Study Questionnaire, so the results are unweighted. Because few data were missing, we conducted a complete case analysis.

## Results

Of the 42700 participants invited to participate in the COVID-19 Questionnaire Study, 23615 were eligible for analysis (response = 55%; Figure 1). Table 1 shows participant sociodemographic characteristics and level of PMH adherence. Participant mean (standard deviation) age at the COVID-19 Baseline Questionnaire was 69.1 (9.5) years.

**Table 1:** Sociodemographic characteristics of the total sample completing the CLSA COVID-19 Exit Questionnaire (N = 23 615), Canada

Characteristics	n	%
Sex^a^		
Female	12 514	52.99
Male	11 101	47.01
Missing	0	N/A
Age, years^b^		
45–54	1075	4.55
55–64	7171	30.37
65–74	8628	36.54
≥ 75	6741	28.55
Missing	0	N/A
Immigrant status^a^		
Yes	3708	15.70
No	19 907	84.30
Missing	0	N/A
Total household income, CAD^c^		
< 50 000	5596	25.08
50 000–100 000	8455	37.89
100 000–150 000	4545	20.37
≥ 150 000	3721	16.67
Missing	1298	N/A
Marital status^c^		
Single/never married	1972	8.36
Married/common law	16 587	70.28
Widowed	2280	9.66
Divorced/separated	2761	11.70
Missing	15	N/A
Educational attainment^a^		
Secondary school graduation or less	3388	14.37
Some postsecondary education	1692	7.18
Postsecondary degree/diploma	18 493	78.45
Missing	42	N/A
Racial background^a^		
White	22 967	97.36
Racialized	623	2.64
Missing	25	N/A
Anxiety status^b^^e^		
Positive	1346	5.93
Negative	21 347	94.07
Missing	922	N/A
Depression status^b^^f^		
Positive	4765	20.54
Negative	18 438	79.46
Missing	412	N/A
PMH adherence level^g^		
Low	6038	25.58
Medium	14 413	61.06
High	3155	13.37
Missing	9	N/A

**Abbreviations: **CAD, Canadian dollars; CLSA, Canadian Longitudinal Study on Aging; PHM, public health measure. 

^a^ Measured via the CLSA Baseline Questionnaire, conducted in 2011–2015. 

^b^ Measured via the CLSA COVID-19 Baseline Questionnaire, conducted in 15 April 2020–30 May 2020. 

^c^ Measured via the CLSA Follow-Up 1 Questionnaire, conducted in 2015–2018. 

^d^ Measured via the CLSA COVID-19 Exit Questionnaire, conducted in September–December 2020. 

^e^ Participants with a score of ≥ 10 on the Generalized Anxiety Disorder 7-item screening tool^29^ were considered to have symptoms of anxiety. 

^f^ Participants with a score of ≥ 10 on the 10-item Center for Epidemiologic Studies Depression Scale^28^ were considered to have symptoms of depression. 

^g^ Levels of PMH adherence were created based on the first quartile (low), second and third quartiles (medium) and fourth quartile (high) of the mean of the average PMH adherence score at each time point (i.e. at the time of the COVID-19 Baseline Questionnaire, conducted between 15 April 2020 and 30 May 2020, and each of the three subsequent monthly questionnaires, conducted in July, August and September 2020). 

***Self-reported changes in alcohol consumption and prospective changes in frequency of alcohol consumption and of binge-drinking events***

**Figure 2:**
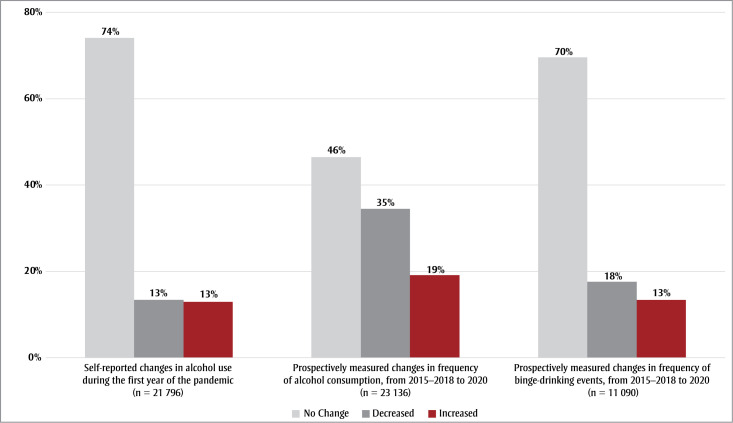
Self-reported changes in alcohol consumption and prospective changes in frequency of alcohol consumption and of binge-drinking events during the COVID-19 pandemic, Canada

**Note: **Data in this figure have been rounded for presentation purposes. 

Of the 21796 participants included in the analysis of self-reported changes in alcohol consumption, 74% (n = 16142) self-reported no change in consumption, while 13% (n = 2921) self-reported decreased consumption and 13% (n = 2733) self-reported increased consumption (Figure 2). Prospective changes in alcohol consumption frequency showed that 46.4% (n = 10744) of 23136 participants had no change in alcohol consumption, while 34.5% (n=7971) decreased consumption and 19.1% (n = 4421) increased consumption. In addition, 69.5% (n = 7710) of 11090 participants showed no change in frequency of binge-drinking events, while 17.6% (n = 1953) showed a decrease and 12.9% (n = 1427) an increase (Figure 2). 

**Table 2:** Cross-tabulation representing the number of participants categorized under self-reported changes in alcohol consumptiona by prospective measures of change in the frequency of alcohol consumption,b Canada

Prospective measure of change in the frequency of alcohol consumption	Self-reported changes in alcohol consumption in 2020, n (%)		
	Decreased (n = 2908)	No change (n = 16 057)	Increased (n = 2731)
Decreased	1708 (59)	5244 (33)	299 (11)
No change	885 (30)	7914 (49)	1250 (46)
Increased	315 (11)	2899 (18)	1182 (43)

**Abbreviation: **CLSA, Canadian Longitudinal Study on Aging. 

**Note:** κ = 0.15. 

^a^ Changes were assessed from the beginning of the pandemic (1 March 2020) to late 2020 (September–December 2020) based on responses to the CLSA COVID-19 Exit Questionnaire. 

^b^ Changes were assessed based on responses to the CLSA Follow-Up 1 Questionnaire (2015–2018) and the CLSA COVID-19 Exit Questionnaire (September–December 2020). 

The agreement between self-reported and prospective changes in frequency of alcohol consumption was low (κ =0.15), possibly because different measurement periods were used. Self-reported changes were assessed from the beginning of the pandemic (1 March 2020); prospective changes were assessed from before the pandemic (2015–2018) (Table 2). We also cannot rule out the possibility that self-reported recall of changes in alcohol may be less valid than prospective measurement.

***Associations between PHM adherence and self-reported and prospective changes in frequency of alcohol consumption and binge-drinking events***

Similar to the unadjusted models, the adjusted results suggest that medium adherence, compared to low, was not associated with lower odds of self-reported decreased alcohol consumption (aOR = 0.97; 95% CI: 0.88–1.07) or with higher odds of self-reported increased alcohol consumption (aOR = 1.00; 95% CI: 0.92–1.11) compared to no change in alcohol consumption. Nor was medium adherence, compared to low, associated with lower odds of decreased frequency of binge-drinking events (aOR = 0.94; 95% CI: 0.83–1.07) or with higher odds of increased frequency of binge-drinking events (aOR = 1.02; 95% CI: 0.89–1.17) (Table 3). However, medium adherence was associated with higher odds of prospectively measured decrease in alcohol consumption frequency (aOR = 1.10; 95% CI: 1.02–1.18) compared to those who reported no change (Table 3). Likewise, high adherence was associated with higher odds of prospectively measured decrease in alcohol consumption frequency (aOR = 1.17; 95% CI: 1.06–1.30) compared to low adherence (Table 3). Wider CIs in high adherence groups may reflect response variability and small sample sizes for prospectively measured changes in frequency of binge-drinking events.

**Table 3:** Associations between PMH adherence and self-reported changes in alcohol consumptiona and prospective changes in frequency of alcohol consumption and of binge-drinking events,b Canada

PMH adherence	Self-reported changes in alcohol consumption^a^						Prospective changes in frequency of alcohol consumption^b^						Prospective changes in frequency of binge-drinking events^b^					
	OR (95% CI) (n = 21 867)			aOR (95% CI)^c^ (n = 20 583)			OR (95% CI) (n = 23 127)			aOR (95% CI)^c^ (n = 21 809)			OR (95% CI) (n = 11 085)			aOR (95% CI)^c^ (n = 10 527)		
	No change	Decrease	Increase	No change	Decrease	Increase	No change	Decrease	Increase	No change	Decrease	Increase	No change	Decrease	Increase	No change	Decrease	Increase
**Low**	Ref.	Ref.	Ref.	Ref.	Ref.	Ref.	Ref.	Ref.	Ref.	Ref.	Ref.	Ref.	Ref.	Ref.	Ref.	Ref.	Ref.	Ref.
**Medium**	Ref.	0.99 (0.90–1.10)	0.89 (0.81–0.97)	Ref.	0.97 (0.88–1.07)	1.00 (0.92–1.11)	Ref.	1.14 (1.06–1.22)	0.92 (0.84–1.00)	Ref.	1.10 (1.02–1.18)	0.96 (0.88–1.04)	Ref.	0.96 (0.86–1.08)	0.96 (0.84–1.09)	Ref.	0.94 (0.83–1.07)	1.02 (0.89–1.17)
**High **	Ref.	1.09 (0.96–1.25)	0.71 (0.61–0.82)	Ref.	1.10 (0.96–1.26)	0.95 (0.81–1.11)	Ref.	1.26 (1.14–1.39)	0.88 (0.78–0.99)	Ref.	1.17 (1.06–1.30)	0.98 (0.87–1.12)	Ref.	0.88 (0.74–1.05)	0.78 (0.64–0.94)	Ref.	0.89 (0.73–1.07)	0.85 (0.68–1.03)

**Abbreviations:** aOR, adjusted odds ratio; CI, confidence interval; CLSA, Canadian Longitudinal Study on Aging; OR, odds ratio; PHM, public health measure; ref., reference. 

^a^ During the first year of the COVID-19 pandemic. Self-reported changes were assessed from the beginning of the pandemic (1 March 2020) to late 2020 (September–December 2020) using the CLSA COVID-19 Exit Questionnaire. 

^b^ From 2015–2018 to 2020. Prospective changes were assessed based on responses to the CLSA Follow-Up 1 Questionnaire (2015–2018) and the CLSA COVID-19 Exit Questionnaire (September–December 2020). 

^c^ Adjusted for sex, age at baseline, household income, marital status, educational attainment, racial background, region of residence and immigrant status. 

***PHM adherence levels***

The proportion of participants with a high PMH adherence was highest at baseline and decreased with time (Supplementary Table 2). Most of the missing observations (6.6% of the included CLSA sample of 23615) were at the first monthly COVID-19 Questionnaire, in July 2020, and fewest of the missing observations were at baseline (0.9% of the CLSA sample of 23615). Missing observations at the second and third COVID-19 monthly questionnaires accounted for 5.6% and 6.2% of the included CLSA sample, respectively. 

Higher proportions of female participants and of participants aged 75 years and older exhibited higher PHM adherence levels (Supplementary Table 3). Higher proportions of individuals at lower household income status exhibited higher adherence levels, while higher proportions of those with higher educational attainment had lower adherence levels. (For distributions of sociodemographic characteristics by self-reported and by prospectively measured changes in frequency of alcohol consumption and by prospectively measured changes in frequency of binge-drinking events, see Supplementary Tables 4, 5 and 6, respectively.)

***Effect modification***

Statistically significant interaction terms were not observed between PHM adherence and age, sex, marital status, immigrant status or household income for outcomes of self-reported change in alcohol consumption (Supplementary Tables 7). Age-stratified results showing the associations between PHM adherence and self-reported changes in alcohol consumption by age group are presented in Table 4. Stratified results suggest associations between high PHM adherence and decreased self-reported change in alcohol consumption were significant and stronger among male participants (Supplementary Table 7). In addition, the association between medium and high, compared to low PHM adherence, and prospective changes in alcohol consumption frequency were significant and stronger for females compared to males (Supplementary Table 9). However, no statistically significant interactions with sex were observed. 

**Table 4:** Adjusted multinomial logistic regression models for self-reported changes in alcohol consumption, stratified by age group, Canada

PMH adherence	45–54 years (n = 975) aOR (95% CI)^a^			55–64 years (n = 6519) aOR (95% CI)^a^			65–74 years (n = 7632) aOR (95% CI)^a^			≥ 75 years (n = 5457) aOR (95% CI)^a^		
	No change	Decreased	Increased	No change	Decreased	Increased	No change	Decreased	Increased	No change	Decreased	Increased
Low	Ref.	Ref.	Ref.	Ref.	Ref.	Ref.	Ref.	Ref.	Ref.	Ref.	Ref.	Ref.
Medium	Ref.	1.00 (0.65–1.56)	1.18 (0.87–1.7)	Ref.	0.92 (0.79–1.11)	0.91 (0.79–1.06)	Ref.	1.11 (0.94–1.32)	1.03 (0.88–1.21)	Ref.	0.86 (0.71–1.04)	1.12 (0.8–1.59)
High	Ref.	0.40 (0.07–0.95)	0.54 (0.21–1.1)	Ref.	1.35 (1.01–1.73)	1.04 (0.81–1.35)	Ref.	1.14 (0.9–1.46)	0.84 (0.63–1.06)	Ref.	0.97 (0.75–1.26)	1.22 (0.78–1.84)

**Abbreviations: **aOR, adjusted odds ratio; CI, confidence interval; CLSA, Canadian Longitudinal Study on Aging; PHM, public health measure; ref., reference. 

**Note: **Interaction term p value = 0.85. 

^a^ Adjusted for sex, total household income, marital status, educational attainment, racial background, region of residence and immigrant status. 

## Discussion

During the first 9 months of the COVID-19 pandemic, from March to December 2020, 74% of 21796 CLSA participants self-reported no change in alcohol consumption, while equal proportions (13%) self-reported a decrease and an increase. Similarly, prospective measures found that most participants did not change their alcohol consumption or frequency of binge drinking, and only a small proportion increased either their consumption or binge drinking. Our results also show that medium and high adherence to PHM, in comparison to low adherence, was associated with higher odds of decreased prospective changes in alcohol consumption frequency. Modifiers of associations between PHM adherence and changes in alcohol consumption were not observed.

While several sources have suggested that alcohol sales and consumption increased across Canada since the start of the pandemic,^4^,^5^,^7^,^17^ our study found that less than 20% of participants increased their consumption, regardless of how changes were measured. This inconsistency may be because we evaluated changes in frequencies of alcohol consumption, rather than changes in quantity of alcohol consumed. Increases in alcohol consumption in Canada have been identified across multiple age groups, including younger adults,^17^,^40^ while our study was conducted with adults aged 50 years and older.

Medium and high PHM adherence were associated with higher odds of prospective decreased frequency of alcohol consumption. However, no associations were observed between medium or high PHM adherence and self-reported decreased alcohol consumption or prospective decreased frequency of binge-drinking events. While not consistent with our initial hypothesis, this is consistent with the findings of several studies that reported associations between higher PHM adherence and fewer occasions of alcohol consumption and heavy drinking.^41^-^43^ Consumption may have also decreased because the imposed PHM restrictions limited access to alcohol, reduced opportunities to socialize and increased health prioritization.^4^

Statistics Canada reported that Canadians aged 65 years and older, who accounted for most of the excess deaths and COVID-19-related deaths between April 2020 and mid-May 2021, were more likely to express health concerns.^11^ This supports research suggesting that older people were less likely to adopt negative health behaviours such as alcohol consumption, early in the pandemic.^44^

Trust in public health communications also affected adherence behaviours,^45^ but future studies are needed to understand how such communications affected alcohol consumption in Canada. Overall, the impact of the pandemic on alcohol consumption is not straightforward, and while some did not increase their alcohol intake, a more nuanced consideration is necessary.

While we initially hypothesized that associations between PHM adherence and changes in alcohol consumption may differ by sex, such differences were not observed. This is consistent with mixed results in the literature. Some studies suggest that males had higher alcohol consumption since the start of the pandemic while others reported that females were more likely to increase consumption consumption.^7^,^46^,^47^ Studies also suggest that young and middle-aged adults in the United States increased their alcohol consumption due to boredom and to relieve stress during the pandemic.^40^

While many studies identified differences in changes in alcohol consumption across age groups, they primarily focused on differences between middle-aged and younger people.^40^ Our study comprised middle-aged and older Canadians. As such, differences within this age range may not be noticeable. In addition, we may not have observed any differences between racial backgrounds, as the proportion of White respondents was significantly larger than the proportion of racialized respondents. Similarly, the proportion of non-immigrant participants in our study was much larger than that of immigrant participants, potentially explaining why any differences in immigrant status were not observed. Overall, changes in alcohol consumption and the association with PHM adherence seem to be consistent within this study sample, as we found it to be independent of sex, age, marital status, immigrant status and region of residence.

Nevertheless, because older individuals are more sensitive to the effects of increased alcohol consumption, including increased risk of chronic conditions,^16^ our results highlight the importance of enhancing substance use services, especially because of the relaxation of alcohol sales and consumption regulations since the onset of the pandemic.^48^

***Strengths and limitations***

Data beyond December 2020 were not available, so our assessment only applies to the first 10 months of the pandemic. Unweighted analyses were conducted as sampling weights were unavailable. While nonparametric bootstrapping was used to improve robustness of standard error estimates, generalizability of study results to the broader Canadian population is limited. 

While agreement between self-reported and prospectively measured changes in alcohol consumption was low, differences in measurement periods limited our ability to validate self-reported recall of changes in consumption. While the risk of underreporting is well-recognized, self-reported alcohol consumption and self-reported changes in consumption are widely used in research^4^,^7^,^17^,^49^ while other measures are still being explored. We did not apply correction factors to the self-reported data because there is no consensus on correction methods for adults in Canada aged 55 years and older. Further, since existing literature largely examines self-reported alcohol consumption changes based on recall while assuming it reflects true changes, we were able to examine prospective measures of changes in alcohol consumption frequency using the longitudinal data available. The results of this study suggest that self-reported changes may not reflect true changes in alcohol consumption. 

Pre-pandemic frequencies of alcohol consumption and binge drinking were obtained from the CLSA FUP1 Questionnaire, conducted between 2015 and 2018, which may not reflect alcohol consumption behaviours immediately before the pandemic. While changes in frequencies of alcohol consumption were examined, information on quantities of alcohol consumed was not collected. Further, since our analysis included only individuals who reported consuming alcohol before the pandemic, our findings do not apply to the participants who reported never consuming alcohol before the pandemic. As such, findings are not generalizable to individuals who initiated alcohol consumption during the pandemic. 

The PHM adherence score developed by De Rubeis et al.^27^ facilitated analysis of adherence over the first 10 months of the pandemic. However, responses to the questionnaire prompts would be subject to recall bias and social desirability bias,^50^ and scores may not reflect true adherence levels. 

While depression and anxiety may affect alcohol consumption, detailed analysis was beyond the scope of this study. 

Lastly, the study sample included few racialized participants and excluded people who were institutionalized, living in the territories, on First Nations reserves and other First Nations settlements, or who were not fluent in either English or French, limiting generalizability to broader, linguistically diverse and Indigenous populations who were differentially impacted by the pandemic.^1^

## Conclusion

We examined associations between PHM adherence and self-reported changes in alcohol consumption and prospective changes in frequencies of alcohol consumption and binge-drinking events from before the start of the COVID-19 pandemic to the end of the first year of the pandemic in Canada. Our findings suggest that high PHM adherence was associated with higher odds of prospective decrease in frequency of alcohol consumption but not with decreased frequency of binge-drinking events or self-reported decreased alcohol consumption. We found no evidence of associations between PHM adherence and increased alcohol consumption early in the pandemic, potentially reflecting health prioritization, healthier behaviours, barriers to purchasing alcohol and less socializing by older Canadians. Because alcohol consumption is an important public health risk factor, more research is needed to understand the impact of public health crises and related measures on alcohol consumption by middle-aged and older adults in Canada.

## Acknowledgements

This research was conducted using the CLSA Baseline Tracking Dataset version 4.0, Baseline Comprehensive Dataset version 7.0, Follow-Up 1 Tracking Dataset version 2.3, Follow-Up 1 Comprehensive Dataset version 3.2, COVID 19 Questionnaire Dataset version 1.0 under Application 2201020. The CLSA is led by Drs. Parminder Raina, Christina Wolfson and Susan Kirkland.

## Funding

Funding for this study was obtained from the Public Health Agency of Canada (PHAC) and the Canadian Institutes for Health Research (CIHR) (grant: CIHR PJT-178394). This research was made possible through the Operating Grants Funding Program, Grant 840241 from the Cancer Research Society in partnership with the Canadian Institutes of Health Research – Institute of Cancer Research (CIHR-ICR; Grant CRP-178672) awarded to L. Anderson. Funding for support of the CLSA COVID-19 questionnaire-based study is provided by the Juravinski Research Institute, Faculty of Health Sciences, McMaster University, the Provost Fund from McMaster University, the McMaster Institute for Research on Aging, PHAC/CIHR (grant reference CMO 174125) and the Government of Nova Scotia.

This research was made possible using the data/biospecimens collected by the CLSA. Funding for the CLSA is provided by the Government of Canada through the CIHR under grant reference LSA 94473, the Canada Foundation for Innovation and the provinces of British Columbia, Alberta, Manitoba, Ontario, Quebec, Nova Scotia, and Newfoundland and Labrador. 

## Conflicts of interest

MdG is a former Associate Editor-in-Chief of the HPCDP Journal, but recused herself from the review process and editorial decision-making for this article.

The authors declare that they have no competing interests. 

## Authors’ contributions and statement

KP: Conceptualization, analysis, writing—original draft, writing—review and editing.

LEG: Writing—review and editing.

AJ: Writing—review and editing.

VDR: Writing—review and editing.

JK: Writing—review and editing.

MdG: Writing—review and editing.

YJ: Writing—review and editing.

JM: Writing—review and editing.

LNA: Conceptualization, writing—original draft, writing—review and editing.

The content and views expressed in this article are those of the authors and do not necessarily reflect those of the Canadian Longitudinal Study on Aging or the Government of Canada.

## Data availability statement

Data are available from the CLSA (www.clsa-elcv.ca) for researchers who meet the criteria for access to de-identified CLSA data. 
